# The T-box transcription factor, TBX3, is a key substrate of AKT3 in melanomagenesis

**DOI:** 10.18632/oncotarget.2782

**Published:** 2014-12-16

**Authors:** Jade Peres, Shaheen Mowla, Sharon Prince

**Affiliations:** ^1^ Department of Human Biology, Faculty of Health Sciences, University of Cape Town, Observatory, 7925, Cape Town, South Africa

**Keywords:** TBX3, AKT3, melanoma, E-cadherin, T-box factors

## Abstract

The AKT3 signalling pathway plays a critical role in melanoma formation and invasion and components of this signalling cascade are therefore attractive targets for the treatment of malignant melanoma. Recent evidence show that the embryonically important TBX3 transcription factor is upregulated in a subset of melanomas and plays a key role in promoting melanoma formation and invasion, in part by repressing the cell adhesion molecule E-cadherin. We have identified TBX3 as a key substrate of AKT3 in melanomagenesis. Briefly, using site-directed mutagenesis and *in vitro* kinase assays, we have identified the AKT3 target site at serine residue 720 in the TBX3 protein and show that this site is phosphorylated *in vivo*. Importantly, we show by western blotting, immunofluorescence, reporter, migration and invasion assays that the phosphorylation at S720 promotes TBX3 protein stability, nuclear localization, transcriptional repression of E-cadherin, and its role in cell migration and invasion. Our results identify a novel signalling and transcriptional network linking AKT3, TBX3 and E-cadherin during melanoma migration and invasion and reveals TBX3 as a potential target for anti-metastatic therapeutics.

## INTRODUCTION

Melanoma is derived from melanocytes and while its early diagnosis and excision results in a five-year survival rate of 99%, the treatment of metastatic melanoma has been mostly ineffective, with a five-year survival rate of less than 16% [1, 2]. The lack of effective long term treatment regimens for metastatic melanoma patients has necessitated investigations into targeted therapeutics which relies on the identification of genetic alterations underpinning the development of this disease [3]. Based on the Clark model, malignant melanoma progresses along well defined stages starting with the radial growth phase where the tumour is confined to the epidermis followed by the vertical growth phase (VGP) when the cells invade the underlying dermis and progress rapidly to metastasize to other organs [4]. This progression is regulated by a complex network of signalling pathways with the Mitogen-activated protein kinase (MAPK) and Phosphatidylinositol-3-OH (PI3) pathways playing prominent roles [5, 6]. The constitutive activation of the MAPK pathway occurs in approximately 60% of sporadic melanomas and results mostly from mutations in the B-Raf gene with the most common being the BRAFV600E mutation [5]. Inhibition of the oncogenic BRAF protein or its downstream effector MEK with the small molecule inhibitors PLX4032 (Vemurafenib) and GSK1120212 (Trametinib) respectively, have shown some promise but patients invariably develop resistance to these drugs [7, 8]. Although the mechanisms underlying BRAF inhibitor resistance are poorly understood, there is increasing evidence suggesting that the PI3K/AKT signalling pathway contributes to intrinsic resistance through suppressing apoptosis and oncogene induced senescence [9–12]. Constitutive activation of the PI3K pathway occurs in approximately 70% of sporadic melanomas due to the loss of PTEN and/or amplification of AKT3 [5, 13–14]. Combined therapies that block both BRAF and PI3K/AKT signalling have therefore been suggested because it could lead to the reactivation of senescence and elimination of melanoma cells refractory to BRAF inhibition.

There are three AKT kinase family members, AKT1, AKT2 and AKT3, and while their Pleckstrin homology and kinase catalytic domains are highly conserved, they are functionally distinct [15–16]. AKT3 is the predominant isoform in melanomas where it plays a critical role in invasion, metastasis and therapeutic resistance [13, 17]. Furthermore, there is growing evidence that activation of this pathway plays a significant role in concurrently activating the MAPK signalling pathway in melanoma. It is therefore not surprising that AKT3 has been identified as a therapeutic target for treating patients with malignant melanoma and indeed there are AKT inhibitors in clinical trials [18]. However, due to the high degree of structural homology between the AKT isoforms, the development of agents that specifically inhibit individual isoforms remains a challenge. Targeting a direct substrate(s) of AKT3 may therefore hold greater promise in the treatment of melanomas. To date, however, no AKT3 substrates have been identified that mediates its roles in the invasion and metastasis of this disease.

The overexpression of the T-box transcription factor, TBX3, was found to correlate with later stages of melanoma, and like AKT3, TBX3 promotes melanoma formation and invasion by, in part, directly repressing E-cadherin [19–22]. While very little is known about the molecular mechanisms by which TBX3 is up-regulated in melanomas, a recent study has shown that TBX3 transcription is upregulated by BRAFV600E [23]. However, mutations in BRAF alone rarely trigger melanoma and additional genetic events in BRAF-mutant cells, such as activation of AKT3, elicit a fully cancerous phenotype [24]. Indeed, recently it was demonstrated that there is a synergistic co-operation between BRAFV600E and AKT3 in promoting melanoma development and it is therefore possible that TBX3 may also be regulated by AKT3 [25]. This possibility is interesting in light of the overlapping functions of TBX3 and AKT3 in melanomagenesis particularly in the negative regulation of E-cadherin.

Here we show for the first time that TBX3 is a novel direct substrate of AKT3 that mediates its role, in part, in melanoma progression. We demonstrate that AKT phosphorylation of TBX3 at serine 720 is responsible for the overexpression and nuclear localization of TBX3 in advanced melanoma cells and that it enhances the ability of TBX3 to repress E-cadherin and promote migration and invasion.

## RESULTS

### Screening TBX3 status in a panel of melanoma cell lines

**Figure 1:**
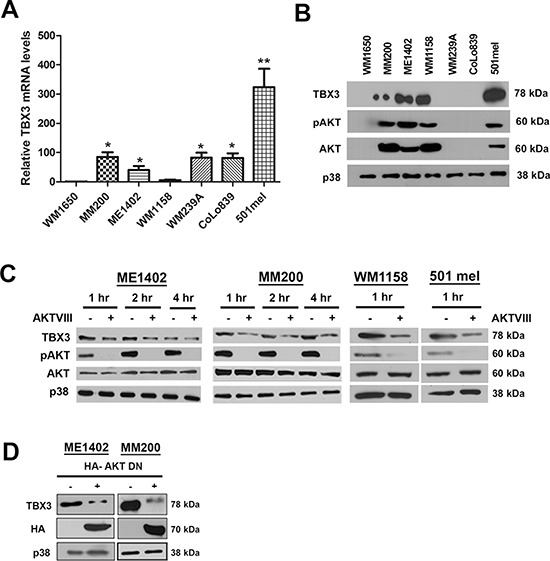
The AKT signalling pathway upregulates TBX3 protein levels in a subset of advanced melanoma cell lines **(A, B)** TBX3 mRNA and protein levels in WM1650 radial growth phase (RGP), MM200 and ME1402 vertical growth phase (VGP), and WM1158, WM239A, CoLo839 and 501mel metastatic melanoma cell lines were analysed by (A) qRT-PCR analyses using primers specific to *TBX3*. For qRT-PCR analyses, the values indicate the mean of three independent experiments ± SEM (*p < 0.05, **p < 0.001) and (B) western blotting using antibodies to TBX3, pAKT and AKT (detects all three AKT isoforms) and p38 was used as a loading control. **(C, D)** Western blotting with antibodies to indicated proteins when the AKT pathway was inhibited by: (C) treating ME1402, MM200, WM1158 and 501 mel cells with either vehicle or 20 μM AKTVIII inhibitor and (D) transiently transfecting the ME1402 and MM200 cells with an HA-tagged AKT dominant negative (DN) construct (100 ng) for 24 hrs.

TBX3 is upregulated in a subset of melanomas and it contributes directly to melanoma formation and invasion [20–22, 26]. To identify the molecular mechanism(s) that upregulate TBX3 in melanoma we screened a panel of human melanoma cell lines for TBX3 mRNA and protein levels. The results showed that in only 3 (MM200, ME1402 and 501mel) out of the 7 cell lines tested there was a direct correlation between TBX3 mRNA and protein levels suggesting that TBX3 may be upregulated at both transcriptional and post translational levels (Figures 1A, 1B).

### The AKT signalling pathway upregulates TBX3 protein levels at a post-transcriptional level in ME1402 and MM200 VGP melanoma cells

To identify the factor(s) that upregulates TBX3 expression levels in melanoma we considered the AKT pathway because it is known to play key roles in melanoma proliferation, migration and invasion and thus overlap with the oncogenic roles identified for TBX3. This possibility was supported by western blot results which showed a direct correlation between pAKT and TBX3 expression (Figure 1B). Furthermore, western blotting shows that TBX3 protein levels were reduced when the AKT pathway was inhibited in the melanoma cells that overexpress TBX3 (Figure 1C, 1D).

**Figure 2:**
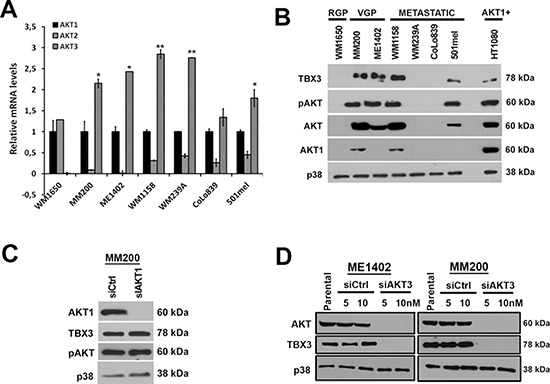
AKT3 is the predominant active isoform in the melanoma cell lines tested and upregulates TBX3 protein levels **(A)** qRT-PCR analyses using primers specific to *AKT1, AKT2* and *AKT3*. In all the indicated cell lines, mRNA levels were firstly normalised to *GUSB* and then expressed relative to *AKT1*. For qRT-PCR analyses, the values indicate the mean of three independent experiments ± SEM (*p < 0.05, **p < 0.001). **(B)** Western blotting with antibodies to TBX3, pAKT, AKT (detects all three AKT isoforms), AKT1 and p38 (used as a loading control). HT1080 (fibrosarcoma) cell extract was used as a positive control for AKT1 expression. **(C–D)** Western blotting with antibodies to indicated proteins when (C) MM200 cells were transiently transfected with siRNA specific to AKT1 (siAKT1) or scrambled control (sictrl) for 48 hrs and (D) ME1402 and MM200 cells were transiently transfected with siRNA specific to AKT3 (siAKT3) or sictrl for 48 hrs.

Of the three AKT isoforms, AKT3 is reported to be the one that is predominantly active in melanoma formation. To explore whether AKT3 was therefore responsible for upregulating TBX3 levels, we firstly performed qRT-PCR to confirm that it was the predominant AKT isoform in our melanoma cell lines. Relative to *AKT1*, *AKT3* was upregulated and *AKT2* downregulated in all the VGP and metastatic melanoma cell lines tested (Figure 2A). Furthermore, AKT1 protein was detectable at low levels in only the MM200 VGP and the WM1158 metastatic melanoma cell lines (Figure 2B) and knocking it down in the MM200 cells had no effect on either the total levels of pAKT or TBX3 (Figure 2C). Importantly, when AKT3 was silenced by siRNA (siAKT3) in ME1402 and MM200 VGP melanoma cells total AKT protein was undetectable and this corresponded with a decrease in TBX3 protein levels (Figure 2D). Together these results suggest that AKT3 was the predominant active isoform in our melanoma cell lines and that it may be responsible for upregulating TBX3 in a subset of these melanoma cell lines.

**Figure 3:**
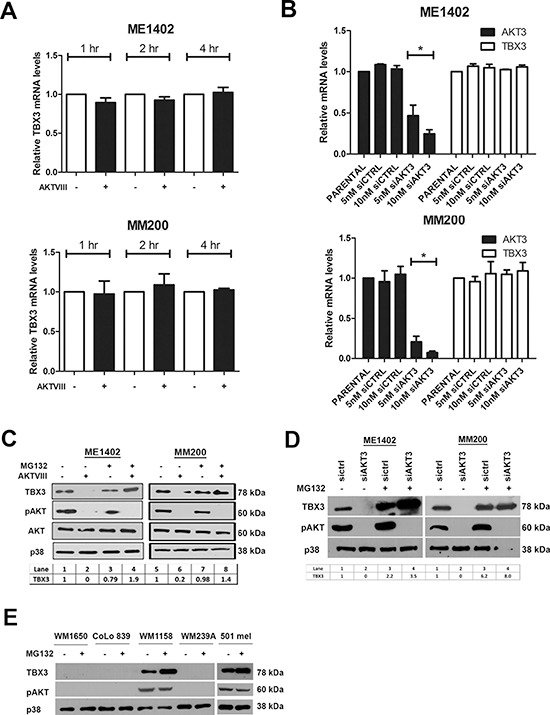
AKT3 upregulates TBX3 protein levels at a post-transcriptional level in melanoma cells **(A, B)** Quantitative RT-PCR was performed on reverse transcribed RNA using primers specific to *TBX3* and *AKT3* and mRNA levels were normalized to *GUSB* and expressed relative to vehicle treated samples. RNA was extracted from ME1402 and MM200 cells (A) treated with either vehicle or 20 μM AKTVIII for 1, 2 and 4 hrs and (B) transiently transfected with siAKT3 or sictrl for 48 hrs. The values indicate the mean of three independent experiments ± SEM (*p < 0.05). **(C, D)** Western blotting of cell extracts from ME1402 and MM200 cells (C) pre-treated with 20 μM MG132 for 15 minutes followed by 20 μM AKTVIII treatment for 1 hr and (D) transiently transfected with siAKT3 or sictrl for 48 hrs and treated with 20 μM MG132 for 15 minutes. Signal intensities were quantified using Image J image analysis software and were normalized to corresponding p38 signals and are shown in tables below western blots in (C) and (D). **(E)** Western blotting of cell extracts from WM1650, CoLo839, WM1158, WM239A and 501 mel cells treated with 20 μM MG132 for 15 minutes. Protein extracts (30 μg) were analysed by SDS-PAGE (8%) and western blotting using antibodies to TBX3 and pAKT and p38 levels was used as a loading control.

Interestingly, TBX3 mRNA levels remained unaltered when the AKT pathway was inhibited with either AKTVIII or siAKT3 (Figures 3A, 3B) and pre-treatment of both melanoma cell lines with the proteasome inhibitor MG132 rescued TBX3 protein levels when the AKT pathway was inhibited (Figure 3C, 3D). MG132 treatment of TBX3 negative melanoma cells could not however rescue TBX3 levels suggesting that TBX3 was repressed in these cells by a mechanism not involving protein degradation by the proteasome 26S (Figure 3E). Together these results suggest that the TBX3 protein is post-translationally modified by AKT3, likely through phosphorylation, which leads to an increase in TBX3 protein stability.

### TBX3 is phosphorylated at S720 *in vitro* and *in vivo*

**Figure 4:**
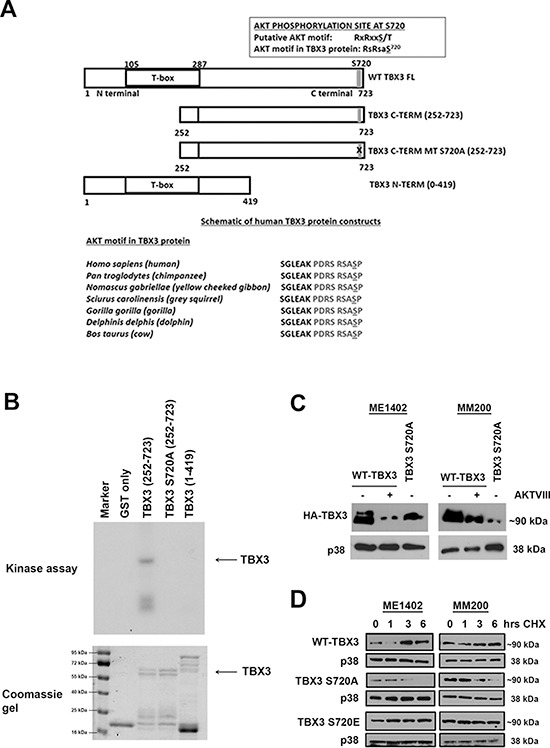
AKT3 phosphorylates TBX3 at serine 720 (S720) and promotes TBX3 protein stability **(A)** Schematic representations of the wild type full length (WT TBX3 FL), C terminal (TBX3 C-TERM (252–723)), C terminal with S720A (TBX3 C-TERM MT S720A (252–723)) and N terminal (TBX3 N-TERM (1–419)) proteins used as substrates in AKT kinase assays. One putative AKT motif, PDRSRSASP, was identified at S720 which was conserved across several species. **(B)** In vitro AKT3 kinase assays were performed using purified GST-TBX3 fusion proteins as substrates in the presence of the recombinant activated AKT3 kinase and [γ-32P] ATP. Kinase assays using the indicated TBX3 proteins are shown in the upper panel after SDS-PAGE and autoradiography. The lower panels show the same gels stained with Coomassie Blue indicating that comparable amounts of protein were used in each assay. **(C)** Mutating the AKT kinase site at Ser-720 affects the phosphorylation of TBX3 *in vivo*. HA-tagged TBX3, or the TBX3 S720A mutant were expressed in ME1402 and MM200 cells treated with either vehicle or 20 μM AKTVIII for 1 hr post transfection and the phosphorylation status of TBX3 was analysed by 7.5% SDS-PAGE and by western blotting using an anti-HA antibody. **(D)** The TBX3 S720A mutant displays a reduced half-life compared to WT TBX3 and the TBX3 S720E mutant. ME1402 and MM200 cells transiently transfected with vectors expressing HA-tagged TBX3 proteins as indicated, were incubated 48 hr post-transfection with 30 μg/ml cycloheximide (CHX) for the indicated times to block de novo protein synthesis. To accurately detect total levels of the TBX3 protein as a single band, cell lysates were analysed on a 10% SDS-PAGE and by western blotting with anti-HA antibodies.

We next examined the TBX3 protein sequence for potential RxRxxS/T AKT phosphorylation sites, using an online motif database [27] and one motif was identified at serine residue 720 which was conserved across several species (Figure 4A). To determine if this site is phosphorylated by AKT3 it was mutated to alanine by site-directed mutagenesis and the protein expressed with a GST tag which was used as a substrate for an *in vitro* AKT3 kinase assay. In this assay the GST-TBX3 fusion proteins were expressed as either N-terminal (1–419) or C-terminal (252–723) proteins and GST alone was used as a negative control (Figure 4A). The results indicate that only the WT GST-TBX3 fusion protein (252–723) was phosphorylated by AKT3 and that the S720A mutation abolished this phosphorylation (Figure 4B). These results show that TBX3 is indeed phosphorylated by the AKT3 kinase at Ser-720 *in vitro*. To test whether this site is also phosphorylated *in vivo*, ME1402 and MM200 VGP melanoma cells were transfected with vectors expressing either HA-tagged WT-TBX3 or a TBX3 S720A mutant in and their phosphorylation status was compared by western blot analyses (Figure 4C). Whereas the WT-TBX3 protein was detected as a double band, the TBX3 S720A mutant was detected as a single band which migrated with the lower WT-TBX3 band. In addition, when the cells transfected with WT-TBX3 was pretreated with an AKT inhibitor, the top WT-TBX3 band was lost. These results suggest that serine residue 720 is a target for *in vivo* phosphorylation and that the top band seen for WT-TBX3 may represent phosphorylated protein since the AKT signalling pathway is constitutively active in these cells.

### Phosphorylation at S720 leads to increase in TBX3 protein stability and nuclear localization

**Figure 5:**
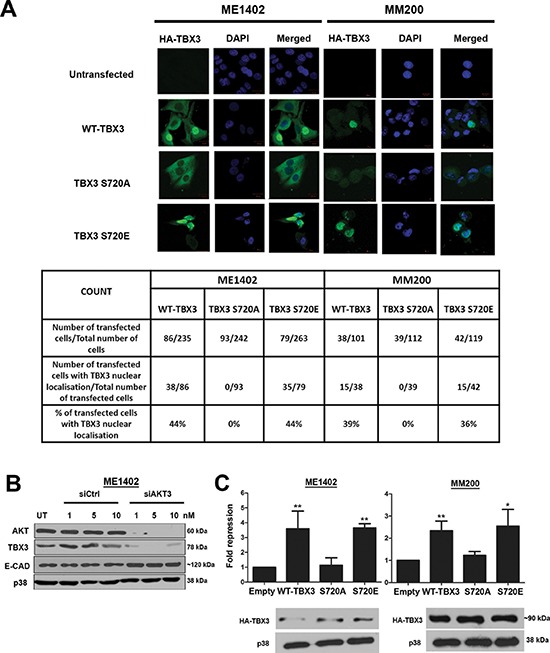
Phosphorylation by AKT3 promotes TBX3 nuclear localization and transcriptional repression of *E-cadherin* **(A)** AKT3 phosphorylation induces nuclear translocation of TBX3. Cells were transfected as described in Figure 4D and then analysed 48 hrs later by immunofluorescence using an anti-HA antibody. Images were captured at 40X magnification using confocal microscopy. Table shows quantitative analyses of five different fields of view at 20X magnification. **(B)** Inverse correlation between TBX3 and E-cadherin (E-cad) protein levels in response to silencing AKT3. Protein extracts from ME1402 cells transiently transfected with siAKT3 or sictrl for 48 hrs were analysed by SDS-PAGE (8%) and western blotting using antibodies to the indicated proteins. **(C)** AKT3 phosphorylation enhances the ability of TBX3 to transcriptionally repress E-cadherin. Cells were transfected as in (A) above together with the *E-cadherin* promoter-luciferase reporter (500 ng). Promoter luciferase activity is indicated as fold repression which represents the ratio of the luciferase activity generated by the pCMV empty vector (without TBX3) to that obtained in the presence of pCMV-TBX3 or TBX3 mutants. *Lower panel*: Western blotting shows equal expression of HA-tagged TBX3 constructs. p38 was used as a loading control.

To determine the functional consequences of TBX3 phosphorylation at S720 we next investigated the effect of phosphorylation of this site on TBX3 protein stability and subcellular localization. To this end, the AKT3 target site was mutated to either alanine (A), to abolish phosphorylation or glutamic acid (E), to mimic phosphorylation and the ME1402 and MM200 VGP melanoma cells were transiently transfected with either WT-TBX3, TBX3 S720A or TBX3 S720E mutant HA-expressing constructs. The transfected cells were treated with cycloheximide, a drug that blocks *de novo* protein synthesis, over a period of 6 hours and protein analysed by western blotting using an anti-HA antibody (Figure 4D). Whereas the TBX3 S720A mutant protein was unstable with a dramatic decrease in protein levels at 6 hours, levels of the pseudo-phosphorylated TBX3 S720E mutant and WT TBX3 proteins either remained unchanged or accumulated at this time point (Figure 4D). This finding implicates AKT3 as a positive regulator of TBX3 protein stability. To establish whether phosphorylation at S720 affected the subcellular localization of TBX3, the VGP melanoma cells were transfected as described above and processed for immunofluorescence with an antibody to HA and the cells visualized by confocal microscopy (Figure 5A). Interestingly whereas on average 40% of cells transfected with WT-TBX3 and TBX3 S720E exhibited nuclear localization of the protein, the TBX3 S720A mutant was exclusively localized to the cytoplasm in all cells transfected with this construct. Taken together the above results show that phosphorylation at S720 regulates TBX3 protein stability and subcellular localization.

### Phosphorylation of S720 enhances the ability of TBX3 to repress the *E-cadherin* promoter and promote cell migration and invasion

Taken together; the above results suggest that TBX3 may be playing a role in the AKT signalling pathway through repressing specific target genes and therefore we next tested whether AKT phosphorylation of TBX3 at S720 enhances its ability to repress transcription of its target genes. *E-cadherin* is a known TBX3 target [20] and its expression has been shown to be inhibited by the AKT pathway in melanoma cells [19] suggesting that AKT-induced phosphorylation of TBX3 may be required to inhibit E-cadherin levels in these cells. We therefore tested this by investigating whether there was an inverse correlation between TBX3 and E-cadherin protein levels in ME1402 VGP melanoma cells in which the AKT pathway was inhibited (Figure 5B). Indeed, there was a substantial increase in E-cadherin protein levels which correlated inversely with the decrease in TBX3 protein levels. To investigate the possibility that AKT phosphorylation potentiates the ability of TBX3 to repress the transcription of the *E-cadherin* gene, we next compared the transcriptional repression of the *E-cadherin* promoter by WT TBX3 and the TBX3 S720A and TBX3 S720E mutants. The results indicate that whereas the WT-TBX3 and TBX3 S720E repressed the *E-cadherin* promoter, abolishing the phosphorylation of the AKT target site (TBX3 S720A) led to an abrogation of this repression (Figure 5C). Equal protein expression was observed for all three constructs tested in the luciferase assays. Taken together these results suggest that phosphorylation of S720 does indeed play a role in regulating the transcriptional activity of TBX3 on the *E-cadherin* promoter in response to AKT signalling.

**Figure 6:**
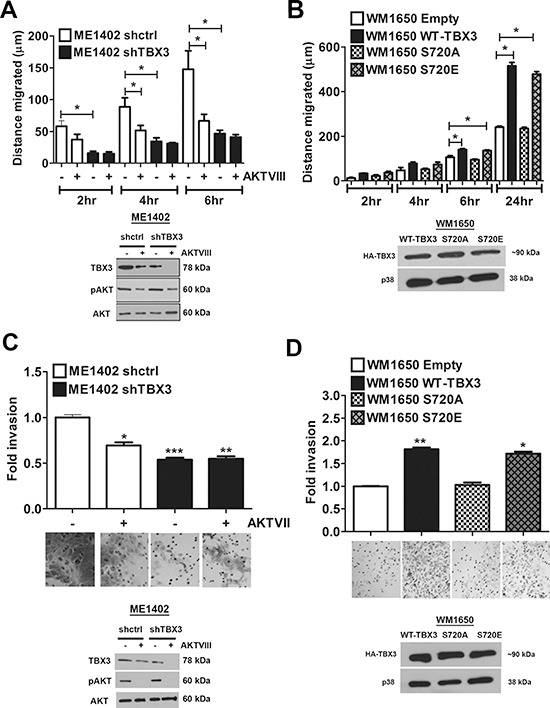
Phosphorylation of TBX3 by AKT3 promotes migration and invasion **(A, C)** TBX3 promotes migration and invasion in ME1402 melanoma cells downstream of AKT signalling. *Upper panel*: (A) Migration or (C) invasion of ME1402 shctrl and shTBX3 cells treated with or without 20 μM AKTVIII for 1 hr was measured at the indicated time points using a two-dimensional in vitro scratch motility assay and a Matrigel invasion chambers based assay. **(A, C)** *Lower panel*: Western blotting shows the expression levels of TBX3, pAKT and AKT in cells treated as for upper panel. **(B, D)** AKT3 phosphorylation of TBX3 promotes migration and invasion of WM1650 RGP melanoma cells. *Upper panel*: Migration of WM1650 RGP melanoma cells transfected with pCMV empty, WT TBX3, TBX3 S720A or S720E for 48 hrs was measured at the indicated time points using (B) a two-dimensional *in vitro* scratch motility assay and (C) a Matrigel invasion chambers based assay. **(B, D)** *Lower panel*: Western blotting shows equal expression of HA-tagged TBX3 constructs. p38 was used as a loading control. The values for all luciferase, migration and invasion assays indicate the mean of three independent experiments ± SEM (*p < 0.05; **p < 0.003, ***p < 0.0002).

Since TBX3 and the AKT pathway both positively impact on cell migration [6, 21–22, 28–30], the possibility that TBX3 may be downstream of the AKT pathway in promoting cell migration was next examined. To this end, ME1402 shctrl and shTBX3 cells were treated with and without AKTVIII and their motility was measured using the two-dimensional scratch (wound) assay. Consistent with previous observations [20–23], knocking down TBX3 severely hampered the migratory ability of the ME1402 cells and, as expected, inhibition of the AKT pathway reduced the migration of ME1402 shctrl cells (Figure 6A). Importantly, when the shTBX3 cells were treated with the AKT inhibitor, there was no significant difference in their migration compared to untreated shTBX3 cells confirming that TBX3 is probably downstream of AKT. To investigate the possibility that AKT phosphorylation enhances the ability of TBX3 to promote migration we next compared the impact of WT TBX3 and the TBX3 S720A and TBX3 S720E mutants on the migratory ability of the WM1650 RGP melanoma cells using an *in vitro* cell motility assay. Figure 6B shows that while WT-TBX3 and TBX3 S720E promoted cell migration, TBX3 S720A transfected cells migrated approximately 50% slower than its counterparts after 24 hrs. When experiments were performed under the same conditions but using Matrigel invasion chambers, AKT signalling was confirmed to be important for the invasive ability of these cells and TBX3 was found to be a key mediator downstream of it (Figure 6C, 6D). These results show that phosphorylation of S720 enhances the ability of TBX3 to promote cell migration and invasion.

## DISCUSSION

Malignant melanoma is an extremely aggressive skin cancer that progresses and metastasizes rapidly and is insensitive to conventional chemo- and radio-therapy. Targeted therapeutics that inhibit the activities of specific genes or signalling pathways involved in the development of this disease are therefore highly desirable. The AKT3 signalling pathway is constitutively active in ~70% of advanced-stage melanomas and components of this pathway represent potential therapeutic targets because it plays a pivotal role in melanoma progression by, in part, repressing the cell adhesion molecule E-cadherin [17, 29]. However, very little is known about the downstream mediators of the AKT3 pathway in this process and in particular the AKT3 substrate(s) responsible for mediating its repressive effects on E-cadherin. Here we describe that AKT3 directly phosphorylates the T-box transcription factor TBX3 which leads to increased TBX3 levels and nuclear localization and an enhancement of its ability to repress E-cadherin and promote migration and invasion. These results show that the AKT/TBX3/E-cadherin axis contributes to melanoma invasion and metastasis and identifies TBX3 as a component of the AKT3 pathway that could be targeted in the treatment of advanced melanomas.

The phosphotidylinositol-3-OH kinase-AKT (PI3K-AKT) pathway is a critical driver of melanoma progression where it plays important roles in promoting cell survival, proliferation, migration, invasion and anti-apoptotic signalling [31]. While AKT3 substrates involved in mediating its impact on proliferation, apoptosis and chemo-resistance in melanoma have been identified, our study is the first to reveal a direct substrate involved in AKT3-induced melanoma migration. AKT3 can promote cell proliferation by phosphorylating GSK3β at Ser-9 resulting in inhibition of its enzymatic activity leading to an increase in cyclin D levels [32–34]. It can also prevent melanoma cell apoptosis by phosphorylating and activating PRAS40 which inhibits caspase-3/7 [17, 35–36]. Furthermore, the PI3K-AKT signalling cascade is known to contribute to melanoma migration and invasion by repressing E-cadherin [13, 37]. Interestingly, the transcription factors Snail, Slug and TBX3 can directly repress *E-cadherin* but only TBX3 contains an AKT consensus motif [20, 38–40]. We show that phosphorylation of TBX3 by AKT at serine 720 enhances its ability to repress *E-cadherin* to promote melanoma migration. However, whether additional mechanisms exist by which AKT represses E-cadherin in melanomas are not known. For example, AKT induces Snail and Zeb2 expression via the NFkB pathway leading to E-cadherin repression in squamous cell carcinoma cell lines [19] and it will be interesting to investigate if this is also the case in melanoma.

The overexpression of TBX3 in a subset of melanomas is required for melanoma cell migration and invasion but very little is known about the mechanism(s) responsible for upregulating TBX3 in this cancer [20–22, 26]. Indeed, to the best of our knowledge the study by Boyd et al [23] showing that TBX3 is transcriptionally upregulated by BRAF^V600E^ in melanoma is the only report to date. Interestingly, a comparison of TBX3 protein and mRNA levels in a panel of melanoma cell lines in our study suggested that TBX3 may be upregulated via both transcriptional and posttranslational mechanisms. These data are interesting in light of the report by Niwa et al. [41] that the PI3K/AKT pathway upregulates Tbx3 via the cytokine leukemia inhibitory factor in order to maintain self-renewal in mouse embryonic stem cells. Together this suggests that the PI3K/AKT pathway may regulate TBX3 via different mechanisms and that this may depend on a developmental versus a cancer context. Furthermore, our data are significant because not much is known about how post-translational modifications of TBX3 regulate its levels and/or function. Interestingly, Yano et al. [42] reported that TBX3 is phosphorylated by the p38 MAPK kinase at SP692 which enhances its ability to transcriptionally repress E-cadherin in COS cells. It is therefore tempting to speculate that the p38 MAPK pathway may also play a role in regulating TBX3 protein levels in melanoma. However, our observations that TBX3 levels are undetectable when AKT3 is silenced suggest that this is not the case. Our data, together with that reported by Boyd et al. [23], suggest that during melanoma development TBX3 expression is transcriptionally regulated by the MAPK pathway and that the stability and oncogenic activity of the TBX3 protein is regulated post-transcriptionally through phosphorylation by AKT3. Together these observations indicate that two pathways critical for melanoma development converge on TBX3 and highlight the importance of TBX3 in melanomagenesis. However, it will be important to determine whether these are the only mechanisms responsible for the role of TBX3 in melanomas and whether AKT3 can lead to an increase in TBX3 levels regardless of BRAF status.

The tumour suppressor phosphatase and tensin homolog (PTEN) is mutated in approximately 40% of melanomas leading to its loss of function and the subsequent upregulation of the PI3K/AKT pathway and an increase in metastasis [43]. There is however also evidence that TBX3 may function upstream of the PI3K/AKT3 signalling cascade by repressing PTEN [44]. In support of this possibility, a microarray conducted by us identified IGF1 and PTEN as possible targets of TBX3 in vertical growth phase melanoma cells. While IGF1, a well-known activator of the PI3K/AKT pathway, appeared to be activated by TBX3, PTEN was shown to be repressed. Together these findings suggest that TBX3 may also be involved in activating the PI3K/AKT3 signalling cascade and thus its own upregulation by this pathway. This autoregulatory positive-feedback loop has also been proposed for TBX3 during development. For example, while Wnt signalling upregulates the expression of Tbx3 in mammary gland development, there is also evidence to suggest that Tbx3 may activate this pathway [45]. This is illustrated by observations that expression of both Wnt10b and the downstream Wnt target Lef1 is lost in the mammary buds of Tbx3 mutant embryos and overexpression of Tbx3 in the flank expanded both the Wnt10b and Lef1 expression domains [46–47].

**Figure 7:**
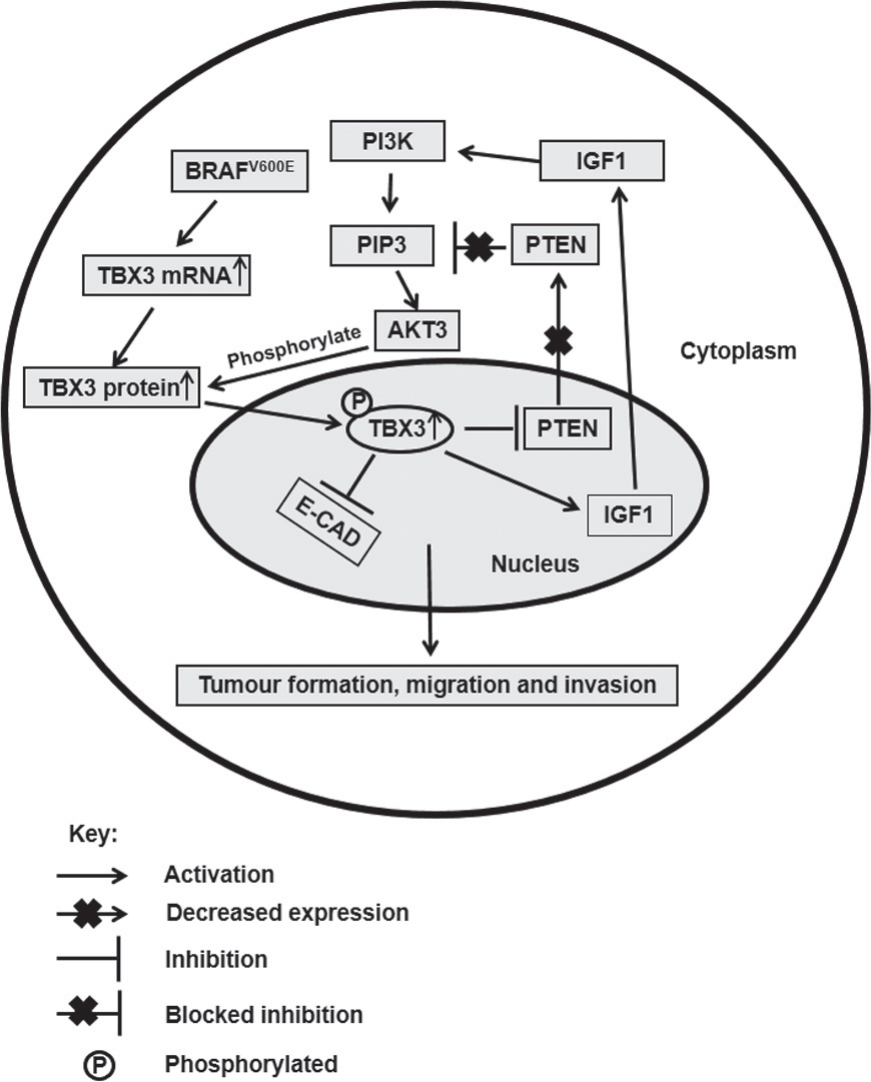
Schematic showing our hypothesis for the role and regulation of TBX3 in advance melanoma TBX3 is upregulated by constitutive activation of both the MAPK and AKT pathways. For example, BRAFV600E transcriptionally upregulates TBX3 and phosphorylation by AKT3 on S720 of the TBX3 protein increases its stability and nuclear localization. Once in the nucleus, TBX3 transcriptionally regulates its target genes, which include repressing E-cadherin and PTEN and activating IGF1. Inhibition of PTEN and activation of IFG1 ensures the constitutive activation of the PI3K/AKT pathway and therefore the upregulation of TBX3. Downregulation of the cell adhesion molecule E-cadherin on the other hand promotes the migration and invasion of advance melanoma cells.

In conclusion, we show that TBX3 is a novel direct substrate of AKT3 that mediates its invasive role, in part, by repressing *E-cadherin* during melanoma progression (Figure 7). Furthermore, we provide a novel mechanism by which TBX3 is upregulated in a subset of advanced melanomas. Our study identifies a signalling and transcriptional network linking AKT, TBX3 and E-cadherin during melanoma migration and invasion and reveals TBX3 as a potential target for novel anti-metastatic therapeutics.

## METHODS

### Plasmids

The wild type (WT) pCMV-TBX3 full length (FL) expression construct was kindly provided by Christine Campbell (Cleveland Clinic Foundation, USA). The human pGEX-TBX3 FL construct was used to generate the human pGEX TBX3 N terminal and PGEX TBX3 C terminal constructs. Point mutations were introduced into the human pCMV-TBX3 or pGEX-TBX3 C terminal cDNA templates by site-directed mutagenesis using the Stratagene QuikChange system. The WT *E-cadherin*-LUC reporter construct was kindly provided by Colin Goding (Ludwig Institute, UK). pCMV5-AKT dominant negative (K179M) (plasmid number 16243, University of Texas MD Anderson Cancer Centre, USA) were acquired from Addgene (http://www.addgene.org, Cambridge, UK).

### Cell culture and treatments

All human melanoma cells were maintained as previously described [21–22]. Prior to treatments, cells were serum starved for 24 hrs. AKT VIII inhibitor (10 μM, Calbiochem, USA) was added to cells for 1 h. MG132 (20 μM, Calbiochem, USA) was added to the cells 1 hr prior to AKT VIII treatments or after siRNA transfections (48 hours later) for 1 hr. Melanoma cells were pre-treated with 30 μg/ml cycloheximide to block *de novo* protein synthesis (Sigma, USA) for 0, 1, 3 and 6 hrs. Control cells were treated with the drug vehicle, DMSO. After drug addition, cells were kept in the dark.

### Transfection assay

Transfections were performed using FuGENE HD (Roche Applied Science) for MM200 cells and Transfectin (Bio-Rad) for ME1402 cells, according to the manufacturers' instructions. For luciferase assays, cells were transfected with 500 ng of the WT E-cadherin-LUC reporter plus 100 ng of the Empty or WT or mutant pCMV TBX3 expression plasmids. The vector pRL-TK was used as an internal control for transfection efficiency (50 ng/transfection). Cells were cultured for 30 h, and extracts were assayed for firefly and Renilla luciferase activity using the dual luciferase assay system (Promega, Madison, WI). Luciferase activities were measured using the Luminoskan Ascent luminometer (Thermo Labsystems, Franklin, MA).

### Small interfering RNA (siRNA)

Suppression of AKT1 and AKT3 expression was achieved by siRNA (small interfering RNA) that specifically targets AKT1 or AKT3 mRNA. The cells were transfected with 10nM anti- AKT1 siRNA (Dharmacon, USA), 5 and 10 nM anti-AKT3 siRNA or a control (non-silencing) (Qiagen, USA), using Lipofectamine 2000 (Invitrogen Life Technology, San Diego, CA, USA) according to the manufacturer's instructions.

### Quantitative real-time PCR (qRT–PCR)

Quantitative real time PCR of cell lines were performed as previously described [48]. Reactions were performed using the primers for human *TBX3* (QT00022484; Qiagen), *GUSB* (QT00046046; Qiagen), *AKT1* (5′-ATGAGCGACGTGGCTATTGTGAAG-3′ and 5′-GAGGCCGTCAGCCACAGT CTGGATG-3′), *AKT2* (5′-TGCTTGAGGCTGTTGGCGACC-3′ and 5′-ATGAATGAGGTGTCTGT CATCAAAGAAGGC-3′) and *AKT3* (5′-ATGAGCGATGTTACCATTGT-3′ and 5′-CAGT CTGTCTGCTACAGCCTGGATA-3′).

### Western blot analysis

Cells were harvested and protein prepared as described previously [48]. Primary antibodies used were as follows: rabbit polyclonal anti-TBX3 (Zymed) and rabbit polyclonal anti-p38 (Sigma, USA), mouse HA monoclonal antibody (62-2, Sigma, USA), rabbit polyclonal anti-phospho AKT (9271), anti- total AKT (9272; detects all three AKT isoforms) and ant-total AKT1 (C73H10) (Cell signalling).

### AKT3 protein kinase assays

Recombinant active AKT3 was obtained from Sigma Aldrich. 500 ng of active AKT3 protein kinase were added to TBX3 recombinant protein in the presence of [γ-32P] ATP (10 μM Ci diluted with 9 μl of 10 μM unlabelled ATP) and incubated for 30 min at 30°C. Following the kinase reaction, beads were washed three times with 1 ml of reaction buffer, and 20 μl of protein denaturing buffer were added. Phosphorylation of TBX3 by the AKT3 kinase was detected by autoradiography after SDS-PAGE gel electrophoresis.

### Immunofluorescence

Cells were grown on coverslip slides, transfected with 100 ng of the WT or mutant pCMV TBX3 expression plasmids. After 48 hrs, the cells were washed with phosphate buffered saline and fixed with 4% paraformaldehyde before permeabilization with 0.2% Triton X-100. Slides were incubated overnight with mouse HA monoclonal antibody (62-2, Sigma, USA) at a dilution of 1:750 and then incubated with Alexa 488 goat anti-mouse IgG (Molecular Probes, Eugene, OR) at a dilution of 1:1000. Cells were incubated with 1 μg/ml DAPI (4′,6-diamidino-2-phenylindole) (Sigma, USA), mounted on a slide and examined by confocal fluorescent microscopy.

### *In vitro* cell migration assay

Cell migration in culture was measured using a two-dimensional *in vitro* scratch motility assay as described previously [21].

### *In vitro* matrigel invasion assay

In brief, transwell inserts with 8-μm pores (BD Biosciences) were coated with Matrigel (272 μg/ml). Approximately 4 × 10^5^ cells were seeded in the upper chambers in 500 μl serum free medium, while 500 μl of medium supplemented with 10% FBS as a chemoattractant was placed in the lower wells. The chambers were incubated at 37°C in a CO^2^ incubator. After 48 hours, the chambers were pulled out, and the non-invading cells on the upper surface were removed with the cotton swab. The cells that invaded to the lower surface of the membrane were fixed in methanol, air dried, and stained with 0.1% crystal violet for 10 minutes. The stained cells were counted in 10 random fields (at ×200 magnification) using a light microscope. Fold invasion was calculated as the number of cells that had passed through the Matrigel-coated membranes relative to the untreated or empty control transfected cells.

### Statistical analysis

Statistical analysis was performed by using the two sample *t*-test (excel) and all graphs were plotted using GraphPad Prism software (GraphPad Prism software, San Diego, CA).

## References

[1] Ferlay J, Bray F, Pisani P, Parkin DM (2004). GLOBOCAN 2002:Cancer incidence, mortality and prevalence worldwide.

[2] González-de Arriba M, Bordel-Gómez MT, Solera JC, Sánchez-Estella J (2013). Primary Dermal Melanoma: A Case Report and a Review of the Literature. Actas Dermosifiliogr.

[3] Coit DG, Andtbacka R, Anker CJ, Bichakjian CK, Carson WE, Daud A, Dilawari RA, Dimaio D, Guild V, Halpern AC, Hodi FS, Kelley MC (2012). Melanoma. J Natl Compr Canc Network.

[4] Miller A, Mihm M (2006). Melanoma. The New England J of Medicine.

[5] Dhawan P, Singh AB, Ellis DL, Richmond A (2002). Constitutive Activation of Akt/Protein Kinase B in Melanoma Leads to Up-Regulation of Nuclear Factor-κB and Tumor Progression. Cancer Research.

[6] Dai DL, Martinka M, Li G (2005). Prognostic significance of activated Akt expression in melanoma: a clinicopathologic study of 292 cases. J. Clin. Oncol.

[7] Chapman PB, Hauschild A, Robert C, Haanen JB, Ascierto P, Larkin J, Dummer R, Garbe C, Testori A, Maio M, Hogg D, Lorigan P (2011). Improved survival with vemurafenib in melanoma with BRAF V600E mutation. N Engl J Med.

[8] Flaherty KT, Robert C, Hersey P, Nathan P, Garbe C, Milhem M, Demidov LV, Hassel JC, Rutkowski P, Mohr P, Dummer R, Trefzer U (2012). Improved survival with MEK inhibition in BRAF-mutated melanoma. N Engl J Med.

[9] Smalley KS, Haass NK, Brafford PA, Lioni M, Flaherty KT, Herlyn M (2006). Multiple signaling pathways must be targeted to overcome drug resistance in cell lines derived from melanoma metastases. Mol Cancer Ther.

[10] Tran MA, Gowda R, Sharma A, Park EJ, Adair J, Kester M, Smith NB, Robertson GP (2008). Targeting V600EB-Raf and Akt3 using nanoliposomal-small interfering RNA inhibits cutaneous melanocytic lesion development. Cancer Res.

[11] Paraiso KH, Xiang Y, Rebecca VW, Abel EV, Chen YA, Munko AC, Wood E, Fedorenko IV, Sondak VK, Anderson AR, Ribas A, Palma MD (2011). PTEN loss confers BRAF inhibitor resistance to melanoma cells through the suppression of BIM expression. Cancer Res.

[12] Vredeveld LC, Possik PA, Smit MA, Meissl K, Michaloglou C, Horlings HM, Ajouaou A, Kortman PC, Dankort D, McMahon M, Mooi WJ, Peeper DS (2012). Abrogation of BRAFV600E-induced senescence by PI3K pathway activation contributes to melanomagenesis. Genes Dev.

[13] Stahl JM, Sharma A, Cheung M, Zimmerman M, Cheng JQ, Bosenberg MW, Kester M, Sandirasegarane L, Robertson GP (2004). Deregulated Akt3 activity promotes development of malignant melanoma. Cancer Research.

[14] Chudnovsky Y, Khavari PA, Adams AE (2005). Melanoma genetics and the development of rational therapeutics. J Clin Invest.

[15] Brazil DP, Park J, Hemmings BA (2002). PKB binding proteins. Getting in on the Akt. Cell.

[16] Manning BD, Cantley LC (2007). AKT/PKB signaling: navigating downstream. Cell.

[17] Soengas MS, Lowe SW (2003). Apoptosis and melanoma chemoresistance. Oncogene.

[18] Ernst DS, Eisenhauer E, Wainman N, Davis M, Lohmann R, Baetz T, Belanger K, Smylie M (2005). Phase II study of perifosine in previously untreated pateints with metstatic melanoma. Invest. New Drugs.

[19] Julien S, Puig I, Caretti E, Bonaventure J, Nelles L, van Roy F, Dargemont C, de Herreros AG, Bellacosa A, Larue L (2007). Activation of NF-kB by Akt upregulates Snail expression and induces epithelium mesenchyme transition. Oncogene.

[20] Rodriguez M, Aladowicz E, Lanfrancone L, Goding CR (2008). Tbx3 represses E-cadherin expression and enhances melanoma invasiveness. Cancer Research.

[21] Peres J, Davis E, Mowla S, Bennett DC, Li JA, Wansleben S, Prince S (2010). The Highly Homologous T-Box Transcription Factors, TBX2 and TBX3, Have Distinct Roles in the Oncogenic Process. Genes & Cancer.

[22] Peres J, Prince S (2013). The T-box transcription factor, TBX3, is sufficient to promote melanoma formation and invasion. Mol. Cancer.

[23] Boyd SC, Mijatov B, Pupo GM, Tran SL, Gowrishankar K, Shaw HM, Goding CR, Scolyer RA, Mann GJ, Kefford RF, Rizos H, Becker TM (2013). Oncogenic B-RAFV600E Signaling Induces the T-Box3 Transcriptional Repressor to Repress E-Cadherin and Enhance Melanoma Cell Invasion. J. Invest. Dermatol.

[24] Flaherty KT, Puzanov I, Kim KB, Ribas A, McArthur GA, Sosman JA, O'Dwyer PJ, Lee RJ, Grippo JF, Nolop K, Chapman PB (2010). Inhibition of mutated, activated BRAF in metastatic melanoma. The New England J. of medicine.

[25] Cheung M, Sharma A, Madhunapantula SV, Robertson GP (2008). Akt3 and mutant V600E B-Raf cooperate to promote early melanoma development. Cancer Research.

[26] Hoek K, Rimm DL, Williams KR, Zhao H, Ariyan S, Lin A, Kluger HM, Berger AJ, Cheng E, Trombetta ES, Wu T, Niinobe M (2004). Expression profiling reveals novel pathways in the transformation of melanocytes to melanomas. Cancer Research.

[27] Yaffe MB, Leparc GG, Lai J, Obata T, Volinia S, Cantley LC (2001). A motif-based profile scanning approach for genome-wide prediction of signaling pathways. Nat. Biotechnol.

[28] Packer L, Pavey S, Parker A, Stark M, Johansson P, Clarke B, Pollock P, Ringner M, Hayward N (2006). Osteopontin is a downstream effector of the PI3-kinase pathway in melanomas that is inversely correlated with functional PTEN. Carcinogenesis.

[29] Robertson GP (2005). Functional and therapeutic significance of Akt deregulation in malignant melanoma. Cancer Metastasis Rev.

[30] Govindarajan B, Sligh JE, Vincent BJ, Li M, Canter JA, Nickoloff BJ, Rodenburg RJ, Smeitink JA, Oberley L, Zhang Y, Slingerland J, Arnold RS (2007). Overexpression of Akt converts radial growth melanoma to vertical growth melanoma. J. Clin. Invest.

[31] Yajima I, Kumasaka MY, Thang ND, Goto Y, Takeda K, Yamanoshita O, Iida M, Ohgami N, Tamura H, Kawamoto Y, Kato M (2012). RAS/RAF/MEK/ERK and PI3K/PTEN/AKT Signaling in Malignant Melanoma Progression and Therapy. Dermatol. Res. Pract.

[32] Fresno Vara JA, Casado E, de Castro J, Cejas P, Belda-Iniesta C, González-Barón M (2004). PI3K/Akt signalling pathway and cancer. Cancer Treat. Rev.

[33] Ikenoue T, Kanai F, Hikiba Y, Tanaka Y, Imamura J, Ohta M, Jazag A, Guleng B, Asaoka Y, Tateishi K, Kawakami T, Matsumura M (2005). Functional consequences of mutations in a putative Akt phosphorylation motif of B-raf in human cancers. Mol. Carcinog.

[34] Woodgett JR (2005). Recent advances in the protein kinase B signaling pathway. Curr. Opin. Cell Biol.

[35] Madhunapantula SV, Sharma A, Robertson GP (2007). PRAS40 deregulates apoptosis in malignant melanoma. Cancer Research.

[36] Sharma A, Sharma AK, Madhunapantula SV, Desai D, Huh SJ, Mosca P, Amin S, Robertson GP (2009). Targeting Akt3 signaling in malignant melanoma using isoselenocyanates. Clinical Cancer Research.

[37] Khosravi S, Wong RP, Ardekani GS, Zhang G, Martinka M, Ong CJ, Li G (2014). Role of EIF5A2, a downstream target of Akt, in promoting melanoma cell invasion. Br. J. Cancer.

[38] Peinado H, Ballestar E, Esteller M, Cano A (2004). Snail mediates E-cadherin repression by the recruitment of the Sin3A/histone deacetylase 1 (HDAC1)/HDAC2 complex. Mol. Cell Biol.

[39] Bolós V, Peinado H, Pérez-Moreno MA, Fraga MF, Esteller M, Cano A (2003). The transcription factor Slug represses E-cadherin expression and induces epithelial to mesenchymal transitions: a comparison with Snail and E47 repressors. J. Cell Sci.

[40] Oikawa T, Nakamura A, Onishi N, Yamada T, Matsuo K, Saya H (2013). Acquired expression of NFATc1 downregulates E-cadherin and promotes cancer cell invasion. Cancer Research.

[41] Niwa H, Ogawa K, Shimosato D, Adachi K (2009). A parallel circuit of LIF signalling pathways maintains pluripotency of mouse ES cells. Nature.

[42] Yano T, Yamazaki Y, Adachi M, Okawa K, Fort P, Uji M, Tsukita S (2011). Tara up-regulates E-cadherin transcription by binding to the Trio RhoGEF and inhibiting Rac signaling. J. Cell Biol.

[43] Wu H, Goel V, Haluska FG (2003). PTEN signaling pathways in melanoma. Oncogene.

[44] Burgucu D, Guney K, Sahinturk D, Ozbudak IH, Ozel D, Ozbilim G, Yavuzer U (2012). Tbx3 represses PTEN and is over-expressed in head and neck squamous cell carcinoma. BMC Cancer.

[45] Chu EY, Hens J, Andl T, Kairo A, Yamaguchi TP, Brisken C, Glick A, Wysolmerski JJ, Millar SE (2004). Canonical WNT signaling promotes mammary placode development and is essential for initiation of mammary gland morphogenesis. Development.

[46] Cho KW, Kim JY, Song SJ, Farrell E, Eblaghie MC, Kim HJ, Tickle C, Jung HS (2006). Molecular interactions between Tbx3 and Bmp4 and a model for dorsoventral positioning of mammary gland development. PNAS.

[47] Davenport TG, Jerome-Majewska LA, Papaioannou VE (2003). Mammary gland, limb and yolk sac defects in mice lacking Tbx3, the gene mutated in human ulnar mammary syndrome. Development.

[48] Abrahams A, Mowla S, Parker MI, Goding CR, Prince S (2008). UV-mediated regulation of the anti-senescence factor Tbx2. J. Biol. Chem.

